# *GJA1* Expression and Left Atrial Remodeling in the Incidence of Atrial Fibrillation in Patients with Obstructive Sleep Apnea Syndrome

**DOI:** 10.3390/biomedicines9101463

**Published:** 2021-10-13

**Authors:** Yung-Lung Chen, Yung-Che Chen, Ya-Ting Chang, Hui-Ting Wang, Wen-Hao Liu, Shaur-Zheng Chong, Pei-Ting Lin, Po-Yuan Hsu, Mao-Chang Su, Meng-Chih Lin

**Affiliations:** 1 Division of Cardiology, Department of Internal Medicine, Kaohsiung Chang Gung Memorial Hospital, Kaohsiung 833, Taiwan; wenhao@cgmh.org.tw (W.-H.L.); shauz@cgmh.org.tw (S.-Z.C.); r40391132@gmail.com (P.-T.L.); 2 Graduate Institute of Clinical Medical Sciences, College of Medicine, Chang Gung University, Taoyuan 333, Taiwan; 3 School of Medicine, College of Medicine, Chang Gung University, Taoyuan 333, Taiwan; yungchechen@yahoo.com.tw (Y.-C.C.); hsupowan@yahoo.com.tw (P.-Y.H.); maochangsu@yahoo.com.tw (M.-C.S.); 4 Division of Pulmonary & Critical Care Medicine, Department of Internal Medicine, Kaohsiung Chang Gung Memorial Hospital, Kaohsiung 833, Taiwan; 5 Department of Neurology, Kaohsiung Chang Gung Memorial Hospital, Chang Gung University College of Medicine, Kaohsiung 833, Taiwan; emily0606@cgmh.org.tw; 6 Emergency Department, Kaohsiung Chang Gung Memorial Hospital, Kaohsiung 833, Taiwan; gardinea1983@gmail.com

**Keywords:** atrial fibrillation, *GJA1*, left atrial remodeling, nocturnal oxygen desaturation, obstructive sleep apnea syndrome, sleep efficiency

## Abstract

Obstructive sleep apnea syndrome (OSAS) is an important risk factor for atrial fibrillation (AF). *GJA1* gene encoding connexin43, a major protein in cardiac gap junctions, plays a crucial role in the synchronized contraction of the heart and in cardiac arrhythmia. However, little is known regarding the role of *GJA1* expression in the incidence of AF in patients with OSAS. All prospectively enrolled OSAS patients underwent polysomnography, electrocardiography, a 24-h Holter test, and echocardiography. Moderate-to-severe OSAS was defined as an apnea-hypopnea index (AHI) ≥ 15. Exosomes were purified from the plasma of all OSAS patients and incubated in HL-1 cells to investigate the effect of exosomes from patients with and without AF on *GJA1* expression. A total of 129 patients were recruited for this study; 26 were excluded due to an AHI < 15. Of the 103 enrolled patients, 21 had AF, and 82 did not. Multivariate analysis showed diabetes mellitus, lower sleep efficiency, lower left ventricular ejection fraction, and larger left atrial (LA) size were independent predictors of AF occurrence in OSAS patients. The area under the receiver operating characteristic curve for LA with a size ≥38.5 mm for predicting AF occurrence in OSAS patients was 0.795 (95% confidence interval [0.666, 0.925]); *p* < 0.001). *GJA1* expression in HL-1 cells incubated with exosomes from OSAS patients with AF was lower than that with exosomes from patients without AF after controlling for age and sex and was negatively correlated with the AHI and oxygen desaturation index (ODI), especially during the non-rapid eye movement period (NREM) of OSAS patients with AF (all *p* < 0.05). LA size was an independent predictor of AF occurrence in OSAS patients. The AHI and ODI in the NREM period of OSAS patients with AF were negatively correlated with *GJA1* expression in HL-1 cells, which offers a hint that *GJA1* may play a crucial role in the development of AF in patients with OSAS.

## 1. Introduction

Atrial fibrillation (AF) is one of the most common heart diseases. It is a major healthcare problem due to its increasing prevalence and associated thromboembolism, heart failure, frequent hospitalizations, and mortality [1,2]. Obstructive sleep apnea syndrome (OSAS) is the most common type of sleep-disordered breathing (SDB) and is characterized by recurrent episodes of partial or complete pharyngeal obstruction [3]. OSAS has been reported to be related to hypertension, and it increases the risk of AF, heart failure, myocardial infarction, stroke, and even mortality [4,5,6,7,8]. There is a growing body of evidence suggesting that OSAS is considered a risk factor for AF [7]. The incidence of AF in OSAS patients varies from 7.6% to 20% in different studies investigating different populations and using different detection methods [7,9,10]. There are some reports on the relationship between AF and OSAS that show the incidence of AF is associated with OSAS severity, that AF patients with OSAS have a worse prognosis (a higher AF recurrence rate) whether receiving medication or ablation therapy [6,7] and that the treatment strategy (e.g., nasal continuous positive airway pressure) for OSAS may decrease the number of AF attacks [11]. However, little is known about the predictors and pathophysiology of AF occurrence in patients with OSAS, which might be more relevant clinically with regard to both the therapeutic strategy and the clinical outcome. OSAS causes intermittent hypoxemia and hypercapnia, enhances the hyperactivity of the autonomic nervous system, and generates negative intrathoracic pressure [4]. Intermittent hypoxemia and post-apnea reoxygenation lead to increased oxidative stress on the myocardium, which may result in myocardial inflammation and remodeling, thereby offering the substrate for AF [4,12]. In addition, the hyperactivity and imbalance of the autonomic nervous system and the changes in volume and stretch in the atrium caused by increased negative intrathoracic pressure may also have implications for the development of AF [13].

Previous studies have shown that the genes that control inflammation, gap junctions, and atrial fibrosis are associated with the pathophysiologic mechanism of AF [12,14]. Connexin-43 protein is encoded by the *GJA1* gene on chromosome 6 in humans and is expressed by atrial and ventricular cardiomyocytes, vascular smooth muscle cells, endothelial cells, monocytes, and macrophages [15]. Myocardial electrical continuity is maintained by connexins located in gap junctions that maintain low-resistance intercellular coupling. The differences in the expression of connexin-43 cause non-uniform discontinuous conduction and cardiac arrhythmia [16]. Exosomes, membrane-bound vesicles 40–100 nm in diameter, are released from many cell types, such as blood cells, endothelial cells, immunocytes, platelets, and smooth muscle cells [17], and are present in almost all biological fluids [18,19]. The RNAs in exosomes can be taken up by neighboring cells or distant cells when exosomes circulate, and they subsequently modulate recipient cells. The discovery of their function in the genetic exchange between cells has brought increasing attention to exosomes. Since a cardiac sample is impossible to acquire from patients without a surgical indication, we evaluated the effect of exosomes from OSAS patients with and without AF by analyzing their gene expression in the HL-1 cells that contract and retain the phenotypic characteristics of the adult atrial cardiomyocyte [20], in order to understand the possible mechanism of an OSAS influence on the occurrence of AF. Therefore, we hypothesize that LA remodeling, the expression of *GJA1*, and other genes affecting inflammation and fibrosis may be involved in the occurrence of AF in patients with OSAS. This study investigated the predictors of AF occurrence in patients with OSAS and the effect of exosomes from OSAS patients with and without AF on the expression of *GJA1* and other inflammatory and fibrotic genes that are involved in the pathophysiology of AF in HL-1 cells, with the goal of elucidating their association with the occurrence of AF.

## 2. Materials and Methods

### 2.1. Patient Enrollment and Sample Management

Patients with SDB were enrolled in this study from May 2019 through December 2020. Patients with an autoimmune disease, malignancy, acute infection, or age younger than 30 years were excluded from the study. Polysomnography (PSG), electrocardiography (ECG), 24-h Holter test, echocardiographic study, and peripheral blood (PB) sampling were performed after the patients were enrolled in the study. The patients’ PB samples were collected between 8:00 a.m. and 9:00 a.m., and plasma from the PB of patients was used for quantification and purification of exosomes. The patient’s clinical characteristics, including age, sex, and comorbidities such as a history of AF, and PSG, ECG, Holter test, and echocardiographic findings, were analyzed. The definition of heart failure in the patient’s baseline characteristics was based on diagnosis during a previous hospitalization and included heart failure with a reduced and preserved ejection fraction.

### 2.2. PSG Study and Definition of SDB Metrics

All patients enrolled in this study underwent an overnight PSG study. The protocol for the PSG was described in our previous study [21]. Briefly, the overnight PSG study was conducted using a standardized commercial suite (Sandman Elite, Mallinckrodt Inc., St. Louis, MO, USA) set in our sleep center. Sleep parameters were recorded, analyzed, and identified by experienced technicians using standard criteria [22,23,24]. Apnea was defined as a cessation of airflow for ≥10 s. Hypopnea was defined as a reduction of ≥30% in airflow for ≥10 s, accompanied by a fall in arterial oxygen saturation of >4% of the baseline level or associated with an arousal. Moderate-to-severe OSAS was defined as an apnea-hypopnea index (AHI) of ≥15 per hour with associated symptoms, such as excessive daytime sleepiness and witnessed apneas during sleep. The oxygen desaturation index (ODI) was defined as the average number of desaturation episodes with at least a 4% decrease in O2 saturation from the pre-event baseline value per hour of sleep. Sleep efficiency was calculated by dividing the PSG-based total sleep time (TST) by the total time between sleep onset and lights on. The arousal index was defined as the number of arousals per hour.

### 2.3. Definition of AF and Echocardiography Measurement

The ECG and Holter tests of the studied patients were reviewed in detail by 2 electrophysiology (EP) doctors. AF was defined as no discernible repeating P waves using a standard 12-lead ECG recording or a single-lead ECG tracing of ≥30 s, according to the 2020 European Society of Cardiology guideline [13].

Transthoracic 2-dimensional (2D) echocardiography was performed using a Sonos 7500 (Live 3D Echo; Philips Medical Systems, Andover, MA, USA), following our previous echocardiography protocol [20,25]. The left atrial (LA) and ventricular dimensions, aortic root size, and thickness of the interventricular septum and left ventricular posterior wall were determined with conventional M-mode echocardiography. Measurement of the LA diameter was performed using a 2D measure, which is perpendicular to the long axis of the LA posterior wall, inner edge to inner edge, at the level of the aortic sinuses [26,27].

This study was approved by the Institutional Review Board of Chang Gung Memorial Hospital (IRB number: 201801943B0) and conformed to the guidelines set forth by the Declaration of Helsinki. Written informed consent was obtained from the participants before starting the study. The data underlying this article will be shared at reasonable request to the corresponding author.

### 2.4. HL-1 Cell Culture, Exosome Purification, and Treatment of HL-1 Cells

#### 2.4.1. Exosome Isolation, Purification, and Quantification

Exosome isolation, purification, and quantification were conducted as previously reported [28,29,30]. PB samples were drawn from patients with OSAS and collected in vacutainer tubes containing EDTA. Blood samples were processed using a 2-layer Ficoll-Histopaque density gradient centrifugation method (Histopaque 1.077 and 1.119; Sigma Diagnostics, St. Louis, MO, USA), and centrifuged at 400× *g* for 30 min at room temperature; the supernatant was transferred into microcentrifuge tubes and aliquoted and stored at −80 °C. For exosome isolation, the plasma was collected and subjected to differential centrifugation at 4 °C to remove cells and cell debris: 20 min at 500× *g* (twice). The supernatant was collected and incubated with thrombin, mixed gently by flicking the tube and incubating at room temperature for 5 min, and centrifuged at 10,000 rpm for 5 min, with pellets visible at the bottom of the tube. Following this, 250 μL of supernatant was transferred into a new tube, and 63 μL of ExoQuick exosome precipitation solution was added for over 1 h, and then the supernatant was centrifuged at 1500× *g* for 30 min. The supernatant was aspirated, and residual ExoQuick exosome solution was spun down by centrifugation at 1500× *g* for 5 min. Finally, the pellets were resuspended in 100 μL of PBS and stored at −80 °C. The pellet that was considered to be the exosomes was suspended in ice-cold PBS, and the protein concentration was measured using a BCA Protein Assay Kit (Thermo Fisher Scientific, Waltham, MA, USA). Exosomes were quantified using an EXOCET exosome quantitation assay (EXOCET96A-1, System Biosciences, Mountain View, CA, USA), according to the manufacturer’s instructions. Briefly, a standard curve was constructed using exosome standards and test samples of purified exosomes from plasma; they were then run in duplicate with an exosome quantity extrapolated from the standard curve.

#### 2.4.2. HL-1 Cell Culture and Treatment with Exosomes

An HL-1 cardiac muscle cell line was purchased as pooled primary cells from SIGMA (SIGMA-ALDRICH Corp., St. Louis, MO, USA). The HL-1 cells were cultured at 37 °C in an atmosphere of 5% CO_2_ in claycomb basal medium supplemented with 10% FBS (Life Technologies, Carlsbad, CA, USA), 2 mM L-glutamine (Life Technologies), 100 uM norepinephrine (Sigma-Aldrich), and 1% penicillin-streptomycin (Life Technologies) [31]. The cells were then incubated with 10 ug/mL of exosomes derived from OSAS patients with and without AF for 24 h at 37 °C.

### 2.5. Real-Time Quantitative Reverse Transcriptase-Polymerase Chain Reaction Analysis (qRT-PCR) of mRNA Expression in HL-1 Cells

Isolation of HL-1 cells, RNA extraction, cDNA synthesis, and qRT-PCR were performed as previously described [32,33]. Briefly, the 1 μg RNA input for cDNA synthesis was determined by spectrophotometric OD260 measurement, and cDNA was generated with a High-Capacity cDNA Reverse Transcription Kit (Thermo Fisher Scientific, Waltham, MA, USA), following the manufacturer’s protocol. The expression of the 6 inflammation-associated genes was analyzed using the SYBR Green system (Thermo Fisher Scientific, Waltham, MA, USA). SYBR Green Gene Expression Assays for tumor necrosis factor-α (TNF-α), hypoxia-inducible factor-1α (HIF-1α), interleukin (IL)-1β, IL-6, transforming growth factor-β (TGF-β), and *GJA1* were purchased from Applied Biosystems (Waltham, MA, USA). The sequences of the PCR primers are listed in Supplementary Table S1. The inflammation-associated genes of mRNA were normalized to *GAPDH*. All reactions were carried out in a 10 μL final volume containing 5 μL 2X SYBR Green qPCR Master Mix (Thermo Fisher Scientific, Inc., Waltham, MA, USA), 0.2 μL primer sets, 1 μL cDNA, and 3.6 μL nucleotide-free H_2_O. Real-time qRT-PCR was performed in an ABI 7500 Fast Real-Time System (Applied Biosystems), and the PCR cycling parameters were set as follows: 95 °C for 20 s, followed by 40 cycles each at 95 °C for 1 s and then 60 °C for 20 s. The expression levels of the inflammation-associated genes were normalized to the internal control *GAPDH* to obtain the relative threshold cycle (ΔCt). All reactions were run in duplicate.

### 2.6. Statistical Analysis

Descriptive summaries are presented for all patients and for patient subgroups. Quantitative data are reported as percentages, mean ± standard deviation, or median (interquartile range) as an appropriate approach. The differences in categorical variables between OSAS patients with and without AF were analyzed by chi-square or Fisher’s exact test, and the differences in continuous data were compared using Student’s t-test or the Mann–Whitney U test. A binary logistic regression model was used to analyze independent predictors of the occurrence of AF in patients with OSAS. Areas under the receiver operating characteristic curve were constructed for the sensitivity and specificity of LA size to predict the development of AF in patients with OSAS. The differences in mRNA gene expression in HL-1 cells incubated with exosomes derived from OSAS patients with and without AF using the ΔCt values were analyzed using the Mann–Whitney U test. The folds change in mRNA gene expression in HL-1 cells incubated with exosomes derived from OSAS patients with and without AF was determined by 2-ΔΔCt calculation. After controlling for age and sex, partial correlation was used to analyze the relationship between the mRNA gene expression in HL-1 cells and the occurrence of AF. The relationship between mRNA gene expression and SDB metrics in patients with AF was analyzed using Pearson’s correlation. We used SPSS version 17.0 software (SPSS, Chicago, IL, USA) for all statistical analyses.

## 3. Results

### 3.1. Baseline Characteristics of OSAS Patients with and without AF

A total of 129 patients with SDB were prospectively enrolled from May 2019 to December 2021. Twenty-six patients were excluded from the analysis since their AHI was <15 per hour. Of the 103 OSAS patients with an AHI ≥ 15 per hour who were eligible for inclusion in this study, 21 (20.4%) had AF, and 82 (79.6%) did not. A total of 25 (24.3%) of the 103 patients were males, and the average age was 56 ± 10 years. Table 1 presents the differences between OSAS patients with and without AF. Briefly, the patients with AF were significantly older and had higher rates of diabetes mellitus (DM) and heart failure than those without AF (all *p*< 0.05). In addition, patients with AF had a larger LA size and lower left ventricular ejection fraction (LVEF) (all *p* < 0.05). There was no difference in baseline characteristics, including sex, body mass index, waistline, AHI, desaturation index, hypertension, stroke, cognitive impairment, coronary artery disease, cancer, dysthyroidism, and other PSG and echocardiographic parameters between patients with and without AF (Table 1).

Data are expressed as mean ± standard deviation, median (interquartile range) or percentage; 2D-ECHO, 2-dimensional echocardiography; AF, atrial fibrillation; AHI, apnea-hypopnea index; AO, aorta, BMI, body mass index; CAD, coronary artery disease; DM, diabetes mellitus; HF, heart failure; HTN, hypertension; IVS, interventricular septum; LA, left atrium; LVEDD, left ventricular end-diastolic diameter; LVEF, left ventricular ejection fraction; LVESD, left ventricular end-systolic diameter; LVPW, left ventricular posterior wall; LVSV, left ventricular stroke volume; NREM, non-rapid eye movement; ODI, oxygen desaturation index; REM, rapid eye movement; SpO2, oxygen saturation.

### 3.2. Predictors of AF Occurrence in Patients with OSAS

**Figure 1:**
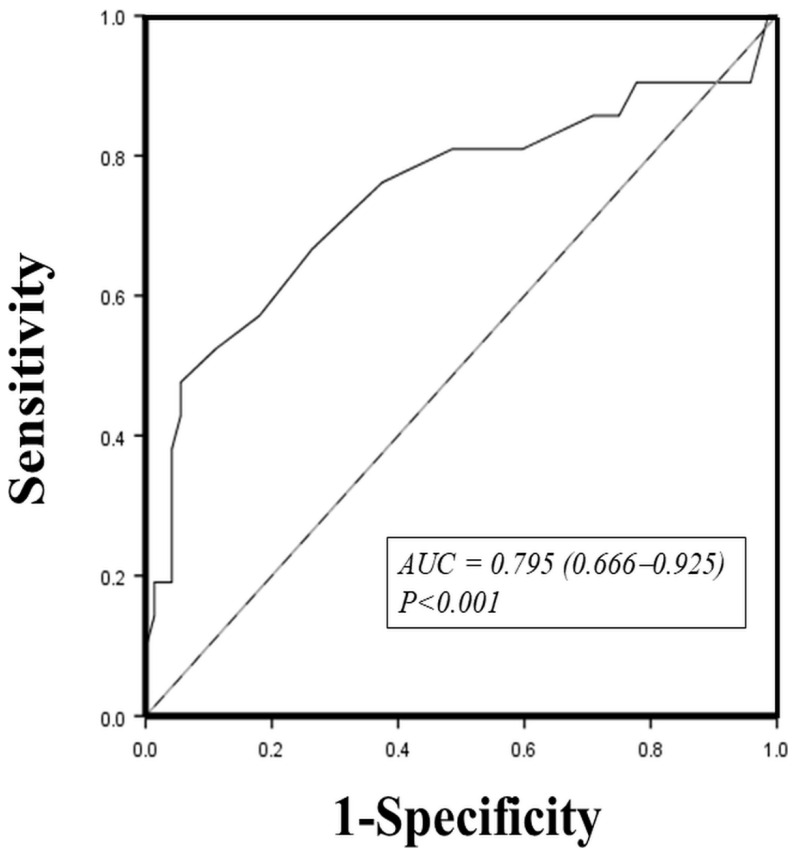
Receiver operating characteristic curve for left atrial size in predicting atrial fibrillation occurrence in patients with obstructive sleep apnea syndrome. AUC: area under the curve.

All relevant variables in Table 1 with a *p*-value < 0.1, including age, DM, sleep efficiency, LA size, and LVEF, were used for the analysis of predictors of AF occurrence. Multivariate stepwise logistic regression analysis revealed that DM (odds ratio (OR): 6.511, 95% confidence interval (CI): 1.140–37.194; *p* = 0.035), lower sleep efficiency (OR: 1.059, 95% CI: 1.007–1.114; *p* = 0.024), lower LVEF (OR: 1.056, 95% CI: 1.002–1.112; *p* = 0.040), and larger LA size (OR: 1.182, 95% CI: 1.051–1.331; *p* = 0.005) were independent predictors of AF occurrence in patients with OSAS (all *p* < 0.05) (Table 2). Since LA size was the most significant predictor of the occurrence of AF, discriminant analysis was performed to identify LA size in predicting the occurrence of AF in patients with OSAS. The area under the receiver operating characteristic curve for a cutoff value ≥ 38.5 mm was 0.795 (95% CI [0.666, 0.925], *p* < 0.001; Figure 1). The sensitivity, specificity, positive predictive value, and negative predictive value for the use of LA with a size ≥ 38.5 mm for predicting AF occurrence in OSAS patients were 66.7%, 73.6%, 63.2%, and 88.4%, respectively.

### 3.3. mRNA Gene Expression in HL-1 Cells Incubated with Exosomes Derived from OSAS Patients with and without AF

**Figure 2:**
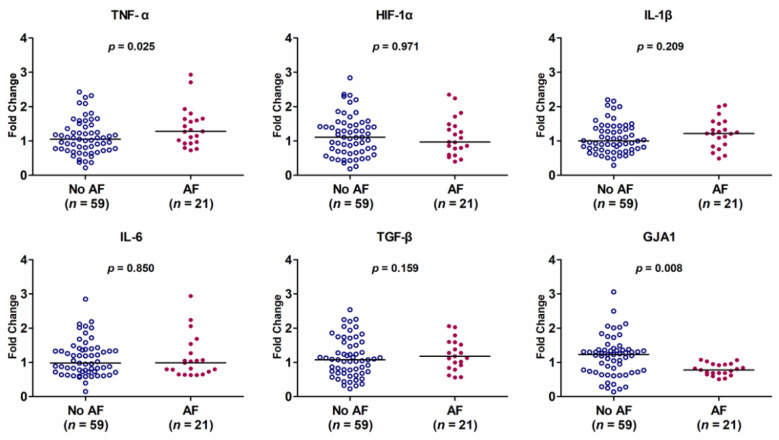
mRNA-level expressions in HL-1 cells incubated with exosomes from obstructive sleep apnea syndrome patients with and without atrial fibrillation (AF). The *y*-axis represents the fold change of the gene expression level in patients with AF compared to patients without AF. The *p*-value analyzed by Mann–Whitney U test indicates statistical significance as evaluated between patients with AF (*n* = 21) and those without AF (*n* = 59) using ΔCt values.

The HL-1 cells were incubated with exosomes derived from 80 studied patients with moderate-to-severe OSAS. Among these patients, 21 had AF, and 59 did not. There was no difference in the exosome particle number between OSAS patients with and without AF. OSAS patients with AF occurrence had lower mRNA-level expressions of *GJA1* and higher mRNA-level expressions of TNF-α (both *p* < 0.05) than OSAS patients without AF occurrence (Figure 2). There was no difference in the mRNA expression of HIF-1α, IL-1β, IL-6, and TGF-β between OSAS patients with and without AF. After controlling for age and sex, the decreased mRNA-level expression of *GJA1* was found to be significantly associated with a higher occurrence of AF in patients with OSAS (r = 0.234, *p* = 0.039).

### 3.4. Correlation between GJA1 Gene Expression in HL-1 Cells and PSG Metrics in OSAS Patients with AF

**Figure 3:**
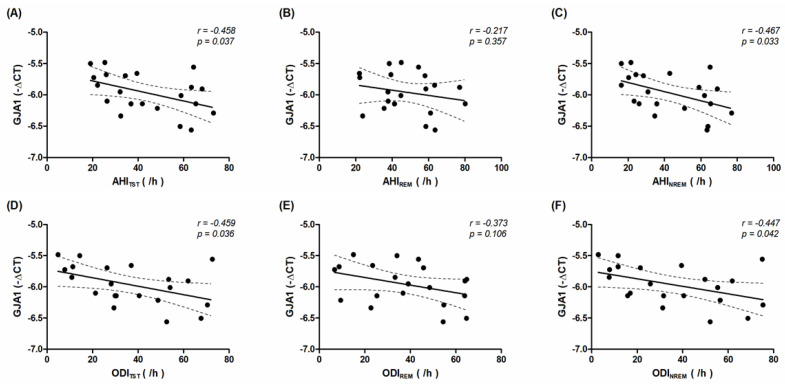
Correlation between *GJA1* gene expression in HL-1 cells and the apnea-hypopnea index (AHI) and oxygen desaturation index (ODI) during total sleep time (TST), rapid eye movement (REM), and non-rapid eye movement (NREM) periods in obstructive sleep apnea syndrome patients with atrial fibrillation (AF). The point in the plot is the observation. The solid line indicates the fitted linear regression line, and its 95% confidence interval is the area enclosed by the dashed curves. The *r*- and *p*-values indicate the correlation between the expression of *GJA1* (expressed as −ΔCt value) and (**A**) AHI_TST_, (**B**) AHI_REM_, (**C**) AHI_NREM_, (**D**) ODI_TST_, (**E**) ODI_REM_, (**F**) ODI_NREM_ (expressed as number per hour).

To investigate the possible impact of OSAS on *GJA1* gene expression in HL-1 cells, we analyzed the correlation between PSG metrics and *GJA1* gene expression after controlling for age and sex. We found there was a negative correlation between *GJA1* gene expression and AHI during TST (r = −0.458, *p* = 0.037), AHI during non-rapid eye movement (NREM) (r = −0.467, *p* = 0.033), ODI during TST (r = −0.459, *p* = 0.036) and ODI during NREM (r = −0.447, *p* = 0.042) (Figure 3). There was no significant correlation between *GJA1* gene expression and AHI during rapid eye movement (REM) and ODI during REM, lowest oxygen desaturation, and the arousal index (all *p* > 0.05).

## 4. Discussion

This study had several important findings. First, OSAS patients with AF had more DM, lower sleep efficiency, a lower LVEF, and a larger LA size than OSAS patients without AF. Second, LA size was the most significant predictor of AF occurrence in OSAS patients, and the cutoff value was 38.5 mm. Third, the mRNA gene expression of *GJA1* was lower, and TNF-α was higher in HL-1 cells incubated with exosomes from OSAS patients with AF than in those incubated with exosomes from OSAS patients without AF. After controlling for age and sex, the gene expression of *GJA1* was still lower in HL-1 cells incubated with exosomes from OSAS patients with AF. Finally, *GJA1* gene expression was negatively correlated with AHI and ODI, especially during the NREM period, in OSAS patients with AF.

Previous studies showed that DM, severity of OSAS, lower sleep efficiency, and heart failure were significantly associated with the incidence of AF in patients with OSAS [7,10,24]. This study confirmed previous findings that DM, lower sleep efficiency, and lower LVEF were independent predictors of AF occurrence in patients with OSAS. One interesting and novel finding in this study was that LA size was also an independent predictor of AF occurrence in patients with OSAS. Hypoxia during sleep may lead to an increased sympathetic tone and oxidative stress, activation of an inflammatory response, and an increase in intrathoracic pressure, preloading of the heart, and tension of the atrial and ventricular wall, which may lead to cardiac remodeling and arrhythmia [4,34,35]. This pathophysiologic mechanism may explain the strong association between LA remodeling and AF occurrence in patients with OSAS.

According to previous studies, LA size is not only associated with AF duration but is also an independent risk factor for AF and a predictor of recurrence of AF after therapy [36]. With an increasing duration and burden of AF, LA structural remodeling has been observed at both the macroscopic and microscopic levels [37]. This might explain why LA size was the most significant predictor for AF occurrence in this study. Most important of all, our study revealed that an LA size < 38.5 mm could be used to exclude AF occurrence in OSAS patients with a high negative predictive value.

Cardiac fibrosis interferes with conduction by impairing intercellular coupling and leads to anisotropic conduction [15]. Inducible deletion of connexin-43 has been reported to be related to the development of cardiac arrhythmia [16]. This study confirmed that exosomes from OSAS patients with AF caused the gene expression of *GJA1* in HL-1 cells to become significantly lower than did exosomes from OSAS patients without AF, independent of age and sex. Further studies should be conducted to evaluate the causative mechanism of decreased *GJA1* expression and AF in patients with OSAS.

AHI is the most common predictor of AF occurrence in patients with OSAS, and the use of continuous positive airway pressure is associated with a significant reduction in the recurrence of AF in patients with OSAS, irrespective of whether they undergo pulmonary vein isolation or medical treatment [38]. Nocturnal oxygen desaturation, which is also an important pathophysiological consequence of OSAS, is an independent risk factor for incident AF in individuals [7]. Our study showed that AHI and DOI were both significantly and negatively correlated with the expression of the *GJA1* gene in OSAS patients with AF, which might offer a possible underlying mechanism for AF occurrence in patients with OSAS. Moreover, a previous study showed that poor sleep quality, in terms of a shorter duration of slow-wave sleep, a factor that was independent of AHI, was associated with AF in an unselected population [24]. This might provide a hint that AHI and ODI in the NREM period rather than in the REM period were significantly associated with the expression of the *GJA1* gene.

Sleep disruption has been linked to increased inflammation mediators, including IL-1, IL-6, TNFα, interferon-γ, and vascular endothelial growth factor in humans [39]. These mediators were also reported to be associated with atrial fibrosis and AF episodes [40]. In this study, the TNFα gene expression in HL-1 cells incubated with exosomes from OSAS patients with AF was higher than that from OSAS patients without AF. TNFα gene expression is associated with age and sex, according to previous studies [41,42]. Old age and male sex are also risk factors for AF occurrence. This may explain why the difference in *TNFα* gene expression in HL-1 cells incubated with exosomes from OSAS patients with and without AF disappeared after adjusting for the factors of age and sex. Further studies should be conducted to investigate whether these inflammatory mediators lead to AF directly or through comorbidities that are associated with AF occurrence. Recent studies have shown upregulation of serum HIF-1α in patients with OSAS [43,44,45]. Continuous 2-month, but not single-night continuous positive airway pressure treatment, could revert the changes in serum HIF-1α protein in patients with OSAS [44,45]. A previous study found significant HIF-1α expression with atrial fibrosis in rats induced with isoproterenol, and atrial fibrosis played a role in the induction and maintenance of AF [46]. Of interest, although the HIF-1α serum protein concentration was higher in patients with OSAS than in patients without OSAS in a previous study, there was no difference in the HIF-1α mRNA expression of peripheral blood leukocytes between patients with and without OSAS [43]. The disparity between HIF-1α expression at mRNA and protein levels may be explained by the possible engagement of posttranslational rather than transcriptional processes, the selection of study subjects, and also the evaluation of cell types. In this study, we compared the mRNA expression in HL-1 cells incubated with exosomes from OSAS patients with and without AF and found there was no difference between the two groups. Further study may be conducted to investigate if there is a difference in the HIF-1α mRNA expression of peripheral blood leukocytes and HIF-1α serum protein levels between OSAS patients with and without AF.

### Limitations

This prospective cohort study had several limitations. First, although we found the *GJA1* expression in HL-1 cells incubated with exosomes from OSAS patients with AF, we did not analyze the correlation between *GJA1* expression in PB from patients and that in HL-1 cells. Even though *GJA1* was expressed in both atrial cardiomyocytes and macrophages, the function of the *GJA1* gene is very different in both types of cells. The roles of connexin-43 in macrophages ranged from migration, antigen-presentation, and phagocytosis to polarization [47]. In contrast, the effect of *GJA1* on cardiomyocytes is essential in non-immune functions in terms of maintaining myocardial electrical continuity. A previous study showed that resident macrophages may play a role in conduction abnormalities by reducing the action potential and aiding early repolarization, which may play a role in conduction abnormalities, including AF and ventricular arrhythmia [48]. Further studies are needed to investigate the relationship between *GJA1* expression in peripheral macrophages and in myocardium-resident macrophages and its role in the development of AF. Second, we did not analyze how RNAs in exosomes from patients modulated the HL-1 cells. Further studies that focus on the effect of exosomes, especially mRNAs, should be conducted to reveal their effect on *GJA1* expression and AF development.

## 5. Conclusions

This prospective cohort study of moderate-to-severe OSAS patients revealed LA size was an independent predictor of the occurrence of AF in patients with OSAS. There was less expression of *GJA1* genes in HL-1 cells incubated with exosomes from OSAS patients, which may play an important role in AF occurrence in patients with OSAS. *GJA1* expression in HL-1 cells was negatively correlated with the AHI and ODI, especially during the NREM period in OSAS patients with AF. Further studies are needed to investigate how exosomes modulate *GJA1* expression and contribute to AF occurrence in OSAS patients.

## Figures and Tables

**Table 1:** Baseline characteristics of obstructive sleep apnea syndrome patients with and without atrial fibrillation.

Variables	No AF (*N* = 82)	AF (*N* = 21)	*p*-Value
Age	56.5 (46–64)	68.6 (53–70)	0.016
Sex (male)	21 (25.6%)	4 (19%)	0.776
Smoking	15 (18.3%)	18 (14.3%)	
BMI	26.3 ± 3.5	26.9 ± 3.4	0.442
Waistline	86.0 ± 7.8	92.0 ± 5.7	0.287
DM	6 (7.3%)	5 (23.8%)	0.029
HTN	31 (37.8%)	10 (47.6%)	0.412
HF	0 (0%)	3 (14.3%)	0.008
Stroke	3 (3.7%)	2 (9.5%)	0.273
CAD	3 (3.7%)	1 (4.8%)	0.824
Cancer	1 (1.2%)	0 (0%)	1.000
Thyroid disorder	1 (1.2%)	1 (4.8%)	0.299
Polysomnography			
Epworth sleepiness scale	8.54 ± 4.7	8.04 ± 5.1	0.673
AHI (per hour)	40.1 (22.6–57.0)	39.5 (26.2–63.5)	0.852
AHI, REM phase (per hour)	48.0 (31.7–63.1)	44.9 (37.7–60.5)	0.859
AHI, NREM phase (per hour)	40.5 (21.5–56.8)	35.8 (23.8–63.7)	0.890
ODI (per hour)	24.9 (10.6–42.7)	30.4 (17.9–53.8)	0.169
ODI, REM phase (per hour)	40.0 (17.2–53.2)	38.0 (22.9–54.6)	0.907
ODI, NREM phase (per hour)	22.1 (9.6–40.8)	31.6 (13.7–56.0)	0.171
Lowest SpO2 (%)	80.0 (72.8–85.3)	78.0 (62.5–88.0)	0.338
Arousal index (per hour)	32.8 (19.9–55.0)	34.3 (14.7–50.1)	0.437
Sleep efficiency (%)	83.3 (73.1–88.7)	79.9 (70.9–85.7)	0.073
2D-ECHO			
AO	32.5 ± 3.8	33.6 ± 4.3	0.244
LA	35.7 ± 4.6	40.3 ± 6.4	<0.001
IVS	11.7 ± 1.7	11.9 ± 2.8	0.714
LVPW	9.3 ± 1.6	9.5 ± 2.0	0.656
LVEDD	47.9 ± 4.4	47.5 ± 7.0	0.780
LVESD	29.1 ± 4.8	31.1 ± 7.1	0.225
LVEF	69.0 ± 9.7	64.1 ± 8.5	0.041

**Table 2:** Univariate and multivariate analysis for predictors of atrial fibrillation in patients with obstructive sleep apnea syndrome.

Variables	Univariate Analysis			Multivariate Analysis		
	OR	95% (CI)	*p*-Value	OR	95% (CI)	*p*-Value
Old age (year)	1.066	1.009–1.125	0.022	1.016	0.946–1.090	0.666
DM	3.958	1.075–14.576	0.039	9.972	1.463–67.988	0.019
Lower sleep efficiency (%)	1.032	0.996–1.068	0.079	1.059	1.007–1.114	0.024
Lower LVEF (%)	1.050	0.993–1.111	0.086	1.062	1.005–1.121	0.033
Larger LA (mm)	1.198	1.073–1.337	0.001	1.219	1.077–1.381	0.005

CI, confidence interval; DM, diabetes mellitus; LA, left atrium; LVEF, left ventricular ejection fraction; OR, odds ratio.

## Data Availability

The data underlying this article will be shared upon reasonable request to the corresponding author.

## Supplementary Materials

The following is available online at https://www.mdpi.com/article/10.3390/biomedicines9101463/s1, Table S1: Primers used for quantitative real-time polymerase chain reaction.

## References

[1] Ahmad Y., Lip G.Y., Lane D.A. (2013). Recent developments in understanding epidemiology and risk determinants of atrial fibrillation as a cause of stroke. Can. J. Cardiol..

[2] Wolf P.A., Dawber T.R., Thomas H.E., Kannel W.B. (1978). Epidemiologic assessment of chronic atrial fibrillation and risk of stroke: The Framingham study. Neurology.

[3] Peppard P.E., Young T., Barnet J.H., Palta M., Hagen E.W., Hla K.M. (2013). Increased prevalence of sleep-disordered breathing in adults. Am. J. Epidemiol..

[4] Somers V.K., White D.P., Amin R., Abraham W.T., Costa F., Culebras A., Daniels S., Floras J.S., Hunt C.E., Olson L.J. (2008). Sleep apnea and cardiovascular disease: An American Heart Association/American College of Cardiology Foundation Scientific Statement from the American Heart Association Council for High Blood Pressure Research Professional Education Committee, Council on Clinical Cardiology, Stroke Council, and Council on Cardiovascular Nursing. In collaboration with the National Heart, Lung, and Blood Institute National Center on Sleep Disorders Research (National Institutes of Health). Circulation.

[5] Pepperell J.C., Ramdassingh-Dow S., Crosthwaite N., Mullins R., Jenkinson C., Stradling J.R., Davies R.J. (2002). Ambulatory blood pressure after therapeutic and subtherapeutic nasal continuous positive airway pressure for obstructive sleep apnoea: A randomised parallel trial. Lancet.

[6] Gami A.S., Pressman G., Caples S.M., Kanagala R., Gard J.J., Davison D.E., Malouf J.F., Ammash N.M., Friedman P.A., Somers V.K. (2004). Association of atrial fibrillation and obstructive sleep apnea. Circulation.

[7] Gami A.S., Hodge D.O., Herges R.M., Olson E.J., Nykodym J., Kara T., Somers V.K. (2007). Obstructive sleep apnea, obesity, and the risk of incident atrial fibrillation. J. Am. Coll. Cardiol..

[8] Yaggi H.K., Concato J., Kernan W.N., Lichtman J.H., Brass L.M., Mohsenin V. (2005). Obstructive sleep apnea as a risk factor for stroke and death. N. Engl. J. Med..

[9] Yeung C., Crinion D., Hammond S., Chacko S., Enriquez A., Redfearn D., Simpson C., Abdollah H., Baranchuk A. (2019). Ambulatory ECG predictors of atrial fibrillation are ineffective in severe sleep apnea. J. Electrocardiol..

[10] Hendrikx T., Sundqvist M., Sandstrom H., Sahlin C., Rohani M., Al-Khalili F., Hornsten R., Blomberg A., Wester P., Rosenqvist M. (2017). Atrial fibrillation among patients under investigation for suspected obstructive sleep apnea. PLoS ONE.

[11] Deng F., Raza A., Guo J. (2018). Treating obstructive sleep apnea with continuous positive airway pressure reduces risk of recurrent atrial fibrillation after catheter ablation: A meta-analysis. Sleep Med..

[12] Burstein B., Nattel S. (2008). Atrial fibrosis: Mechanisms and clinical relevance in atrial fibrillation. J. Am. Coll. Cardiol..

[13] Hindricks G., Potpara T., Dagres N., Arbelo E., Bax J.J., Blomstrom-Lundqvist C., Boriani G., Castella M., Dan G.A., Dilaveris P.E. (2021). 2020 ESC Guidelines for the diagnosis and management of atrial fibrillation developed in collaboration with the European Association for Cardio-Thoracic Surgery (EACTS): The Task Force for the diagnosis and management of atrial fibrillation of the European Society of Cardiology (ESC) Developed with the special contribution of the European Heart Rhythm Association (EHRA) of the ESC. Eur. Heart J..

[14] Thomas S.A., Schuessler R.B., Berul C.I., Beardslee M.A., Beyer E.C., Mendelsohn M.E., Saffitz J.E. (1998). Disparate effects of deficient expression of connexin43 on atrial and ventricular conduction: Evidence for chamber-specific molecular determinants of conduction. Circulation.

[15] Pfenniger A., Chanson M., Kwak B.R. (2013). Connexins in atherosclerosis. Biochim. Biophys. Acta.

[16] van Rijen H.V., Eckardt D., Degen J., Theis M., Ott T., Willecke K., Jongsma H.J., Opthof T., de Bakker J.M. (2004). Slow conduction and enhanced anisotropy increase the propensity for ventricular tachyarrhythmias in adult mice with induced deletion of connexin43. Circulation.

[17] Liao J., Liu R., Yin L., Pu Y. (2014). Expression profiling of exosomal miRNAs derived from human esophageal cancer cells by Solexa high-throughput sequencing. Int. J. Mol. Sci..

[18] Gross J.C., Chaudhary V., Bartscherer K., Boutros M. (2012). Active Wnt proteins are secreted on exosomes. Nat. Cell Biol..

[19] Mathivanan S., Ji H., Simpson R.J. (2010). Exosomes: Extracellular organelles important in intercellular communication. J. Proteom..

[20] Claycomb W.C., Lanson N.A., Stallworth B.S., Egeland D.B., Delcarpio J.B., Bahinski A., Izzo N.J. (1998). HL-1 cells: A cardiac muscle cell line that contracts and retains phenotypic characteristics of the adult cardiomyocyte. Proc. Natl. Acad. Sci. USA.

[21] Chen Y.L., Su M.C., Liu W.H., Wang C.C., Lin M.C., Chen M.C. (2014). Influence and predicting variables of obstructive sleep apnea on cardiac function and remodeling in patients without congestive heart failure. J. Clin. Sleep Med..

[22] Redline S., Budhiraja R., Kapur V., Marcus C.L., Mateika J.H., Mehra R., Parthasarthy S., Somers V.K., Strohl K.P., Sulit L.G. (2007). The scoring of respiratory events in sleep: Reliability and validity. J. Clin. Sleep Med..

[23] Bonnet M.H., Carley D.W., Carskadon M.A., Easton P.A., Guilleminault C., Harper R.M., Hayes B., Hirshkowitz M., Ktonas P.Y., Keenan S. (1992). EEG arousals: Scoring rules and examples: A preliminary report from the Sleep Disorders Atlas Task Force of the American Sleep Disorders Association. Sleep.

[24] Kwon Y., Gharib S.A., Biggs M.L., Jacobs D.R., Alonso A., Duprez D., Lima J., Lin G.M., Soliman E.Z., Mehra R. (2015). Association of sleep characteristics with atrial fibrillation: The Multi-Ethnic Study of Atherosclerosis. Thorax.

[25] Liu W.H., Guo B.F., Chen Y.L., Tsai T.H., Fu M., Chua S., Chen M.C. (2010). Right ventricular outflow tract pacing causes intraventricular dyssynchrony in patients with sick sinus syndrome: A real-time three-dimensional echocardiographic study. J. Am. Soc. Echocardiogr..

[26] Lang R.M., Badano L.P., Mor-Avi V., Afilalo J., Armstrong A., Ernande L., Flachskampf F.A., Foster E., Goldstein S.A., Kuznetsova T. (2015). Recommendations for cardiac chamber quantification by echocardiography in adults: An update from the American Society of Echocardiography and the European Association of Cardiovascular Imaging. Eur. Heart J. Echocardiogr. Imaging.

[27] Chen Y.L., Wang H.T., Chen H.C., Chai H.T., Lee Y.W., Liu W.H. (2021). Localization of right ventricular non-apical lead position: Comparison of three-dimensional echocardiography, computed tomography, and fluoroscopic imaging. J. Int. Med. Res..

[28] Tauro B.J., Greening D.W., Mathias R.A., Ji H., Mathivanan S., Scott A.M., Simpson R.J. (2012). Comparison of ultracentrifugation, density gradient separation, and immunoaffinity capture methods for isolating human colon cancer cell line LIM1863-derived exosomes. Methods.

[29] Lydic T.A., Townsend S., Adda C.G., Collins C., Mathivanan S., Reid G.E. (2015). Rapid and comprehensive ‘shotgun’ lipidome profiling of colorectal cancer cell derived exosomes. Methods.

[30] Huang C., Fisher K.P., Hammer S.S., Navitskaya S., Blanchard G.J., Busik J.V. (2018). Plasma Exosomes Contribute to Microvascular Damage in Diabetic Retinopathy by Activating the Classical Complement Pathway. Diabetes.

[31] Kuznetsov A.V., Javadov S., Sickinger S., Frotschnig S., Grimm M. (2015). H9c2 and HL-1 cells demonstrate distinct features of energy metabolism, mitochondrial function and sensitivity to hypoxia-reoxygenation. Biochim. Biophys. Acta.

[32] Hsu P.Y., Hsi E., Wang T.M., Lin R.T., Liao Y.C., Juo S.H. (2017). MicroRNA let-7g possesses a therapeutic potential for peripheral artery disease. J. Cell Mol. Med..

[33] Nguyen S.V., Claycomb W.C. (1999). Hypoxia regulates the expression of the adrenomedullin and HIF-1 genes in cultured HL-1 cardiomyocytes. Biochem. Biophys. Res. Commun..

[34] Malhotra A., White D.P. (2002). Obstructive sleep apnoea. Lancet.

[35] Fessler H.E. (1997). Heart-lung interactions: Applications in the critically ill. Eur. Respir. J..

[36] Vaziri S.M., Larson M.G., Benjamin E.J., Levy D. (1994). Echocardiographic predictors of nonrheumatic atrial fibrillation. The Framingham Heart Study. Circulation.

[37] Kumar S., Teh A.W., Medi C., Kistler P.M., Morton J.B., Kalman J.M. (2012). Atrial remodeling in varying clinical substrates within beating human hearts: Relevance to atrial fibrillation. Prog. Biophys. Mol. Biol..

[38] Shukla A., Aizer A., Holmes D., Fowler S., Park D.S., Bernstein S., Bernstein N., Chinitz L. (2015). Effect of Obstructive Sleep Apnea Treatment on Atrial Fibrillation Recurrence: A Meta-Analysis. JACC Clin. Electrophysiol..

[39] Guess J., Burch J.B., Ogoussan K., Armstead C.A., Zhang H., Wagner S., Hebert J.R., Wood P., Youngstedt S.D., Hofseth L.J. (2009). Circadian disruption, Per3, and human cytokine secretion. Integr. Cancer Ther..

[40] Wu N., Xu B., Xiang Y., Wu L., Zhang Y., Ma X., Tong S., Shu M., Song Z., Li Y. (2013). Association of inflammatory factors with occurrence and recurrence of atrial fibrillation: A meta-analysis. Int. J. Cardiol..

[41] Bruunsgaard H., Pedersen B.K. (2003). Age-related inflammatory cytokines and disease. Immunol. Allergy Clin. N. Am..

[42] Aomatsu M., Kato T., Kasahara E., Kitagawa S. (2013). Gender difference in tumor necrosis factor-alpha production in human neutrophils stimulated by lipopolysaccharide and interferon-gamma. Biochem. Biophys. Res. Commun..

[43] Gabryelska A., Szmyd B., Szemraj J., Stawski R., Sochal M., Bialasiewicz P. (2020). Patients with obstructive sleep apnea present with chronic upregulation of serum HIF-1alpha protein. J. Clin. Sleep Med..

[44] Lu D., Li N., Yao X., Zhou L. (2017). Potential inflammatory markers in obstructive sleep apnea-hypopnea syndrome. Bosn. J. Basic Med. Sci..

[45] Gabryelska A., Stawski R., Sochal M., Szmyd B., Bialasiewicz P. (2020). Influence of one-night CPAP therapy on the changes of HIF-1alpha protein in OSA patients: A pilot study. J. Sleep Res..

[46] Su F., Zhang W., Chen Y., Ma L., Zhang H., Wang F. (2014). Significance of hypoxia-inducible factor-1alpha expression with atrial fibrosis in rats induced with isoproterenol. Exp. Ther. Med..

[47] Rodjakovic D., Salm L., Beldi G. (2021). Function of Connexin-43 in Macrophages. Int. J. Mol. Sci..

[48] Hulsmans M., Clauss S., Xiao L., Aguirre A.D., King K.R., Hanley A., Hucker W.J., Wulfers E.M., Seemann G., Courties G. (2017). Macrophages Facilitate Electrical Conduction in the Heart. Cell.

