# Supplementary material for: GJA1 Expression and Left Atrial Remodeling in the Incidence of Atrial Fibrillation in Patients with Obstructive Sleep Apnea Syndrome
Source: Biomedicines. 2021 Oct 13;9(10):1463. doi: 10.3390/biomedicines9101463 (PMC8533106; doi:10.3390/biomedicines9101463)
Supplement: Supplementary file 1 [file biomedicines-1398068-supplementary.pdf › biomedicines-1398068-supplementary.pdf]

Supplemental Table S1. Primers used for quantitative real-time polymerase chain reaction

Gene	Primer
TNF-α F	5'- CCC TCA CAC TCA GAT CAT CTT CT -3'
TNF-α R	5'- GCT ACG ACG TGG GCT ACA G -3'
Hif-1α F	5'- ACC TTC ATC GGA AAC TCC AAA G -3'
Hif-1α R	5'- ACT GTT AGG CTC AGG TGA ACT -3'
IL-1β F	5'- GCA ACT GTT CCT GAA CTC AAC T -3'
IL-1β R	5'- ATC TTT TGG GGT CCG TCA ACT -3'
IL-6 F	5'- TAG TCC TTC CTA CCC CAA TTT CC -3'
IL-6 R	5'- TTG GTC CTT AGC CAC TCC TTC -3'
TGF-β F	5'- CTC CCG TGG CTT CTA GTG C -3'
TGF-β R	5'- GCC TTA GTT TGG ACA GGA TCT G -3'
GJA1 F	5'- ACA GCG GTT GAG TCA GCT TG -3'
GJA1 R	5'- GAG AGA TGG GGA AGG ACT TGT -3'
GAPDH F	5'- AGG TCG GTG TGA ACG GAT TTG -3 '
GAPDH R	5'- TGT AGA CCA TGT AGT TGA GGT CA -3 '
